# A laser desorption ionization/matrix‐assisted laser desorption ionization target system applicable for three distinct types of instruments (LinTOF/curved field RTOF, LinTOF/RTOF and QqRTOF) with different performance characteristics from three vendors

**DOI:** 10.1002/rcm.8075

**Published:** 2018-03-26

**Authors:** Edita Rados, Ernst Pittenauer, Johannes Frank, Kurt Varmuza, Günter Allmaier

**Affiliations:** ^1^ Institute of Chemical Technologies and Analytics TU Wien (Vienna University of Technology) Vienna Austria; ^2^ Joint Workshop of the Faculty of Technical Chemistry TU Wien (Vienna University of Technology) Vienna Austria; ^3^ Institute of Statistics and Mathematical Methods in Economics TU Wien (Vienna University of Technology) Vienna Austria

## Abstract

**Rationale:**

We have developed a target system which enables the use of only one target (i.e. target preparation set) for three different laser desorption ionization (LDI)/matrix‐assisted laser desorption ionization (MALDI) mass spectrometric instruments. The focus was on analysing small biomolecules with LDI for future use of the system for the study of meteorite samples (carbonaceous chondrites) using devices with different mass spectrometric performance characteristics.

**Methods:**

Three compounds were selected due to their potential presence in meteoritic chondrites: tryptophan, 2‐deoxy‐d‐ribose and triphenylene. They were prepared (with and without MALDI matrix, i.e. MALDI and LDI) and analysed with three different mass spectrometers (LinTOF/curved field RTOF, LinTOF/RTOF and QqRTOF). The ion sources of two of the instruments were run at high vacuum, and one at intermediate pressure. Two devices used a laser wavelength of 355 nm and one a wavelength of 337 nm.

**Results:**

The developed target system operated smoothly with all devices. Tryptophan, 2‐deoxy‐d‐ribose and triphenylene showed similar desorption/ionization behaviour for all instruments using the LDI mode. Interestingly, protonated tryptophan could be observed only with the LinTOF/curved field RTOF device in LDI and MALDI mode, while sodiated molecules were observed with all three instruments (in both ion modes). Deprotonated tryptophan was almost completely obscured by matrix ions in the MALDI mode whereas LDI yielded abundant deprotonated molecules.

**Conclusions:**

The presented target system allowed successful analyses of the three compounds using instruments from different vendors with only one preparation showing different analyser performance characteristics. The elemental composition with the QqRTOF analyser and the high‐energy 20 keV collision‐induced dissociation fragmentation will be important in identifying unknown compounds in chondrites.

## 1 INTRODUCTION

Analysing small molecules (<800 Da) with matrix‐assisted laser desorption/ionization time‐of‐flight mass spectrometry (MALDI‐TOF‐MS) can be a challenge due to the so‐called ‘chemical noise’ originating mainly from commonly used MALDI matrix molecules.^1^, ^2^ These MALDI matrices produce matrix‐related ions of high intensities in the low *m*/*z* range, which interfere with or even obscure analyte‐related ions.^3^, ^4^, ^5^ By using laser desorption ionization (LDI) instead of MALDI, the chemical noise problem arising from excess MALDI matrix in the low *m*/*z* region can be avoided. LDI is also useful for analytes that cannot be dissolved easily in common solvents that are regularly applied to dissolve standard MALDI matrices. Despite the much higher thermal load transferred to the molecules of interest in LDI, it is often a successful approach. It is often also difficult to select one individual appropriate MALDI matrix for chemically different analytes, because not every matrix works satisfactorily with the selected sample.^5^, ^6^, ^7^ There are further differences in MALDI matrix selection for one given analyte between classical high‐vacuum MALDI (with a TOF‐MS instrument) and intermediate‐pressure MALDI (typically with ion traps, hybrid quadrupole/reflectron TOFs and FT‐ICR‐MS instruments).^8^, ^9^

The UV LDI‐MS method typically requires a higher laser fluence to desorb analyte ions from the target surface than MALDI‐MS. Furthermore the analytes have to exhibit particular structural features (e.g. conjugated systems or aromatic features) fitting the wavelength of the applied UV laser. The resulting excess energy for desorption/ionization produces not only intact ions ([M + H]^+^, [M − H]^−^, [M + Na]^+^, [M + 2Na − H]^+^ ions and M^+•^ radical cations), but also frequently a significant number of in‐source‐generated fragment ions. As different manufacturers of MALDI/LDI instruments usually provide different target plates in terms of format, and surface structures including surface chemistry and fine structure surface only useful for one particular instrument, a serious comparison of one sample preparation is usually not easy. On the other hand, the ability to work on different MALDI/LDI instruments with only one target plate ensures that the sample is always prepared on the same surface and that no additional contamination can occur, which can be the case when using multiple target plates (despite careful cleaning procedures). This approach could be helpful if only small sample amounts are available and thus only one or two preparations, i.e. sample spots, can be made. The switching from one type of ion source (e.g. intermediate pressure to high vacuum or different entry angles of the laser beam or different laser wavelengths) and/or of differently performing analysers can be done in a straightforward manner only if part of the sample is consumed (which is usually the case except perhaps in mass spectrometric imaging).

In this paper we compare the mass spectrometric behaviour of three selected small‐molecule analytes (tryptophan, 2‐deoxy‐d‐ribose and triphenylene) deposited on a standard Waters MALDI‐MS target mounted on a home‐built target adapter/holder using three different instruments (Axima TOF^2^, ultrafleXtreme and Synapt G2) in both LDI and MALDI modes. For this study our goal is the evaluation of the desorption/ionization properties of these model analytes utilizing one target with one preparation (including replicates) on different performing instruments (e.g. different laser wavelength applied in LDI mode, different LDI beam shapes, different laser pulse rates, different ion source pressures, different mass spectrometric accuracy and resolution, and high‐energy versus low‐energy collision‐induced dissociation) with a home‐built target adapter for three instruments. The resulting data obtained by LDI‐MS were compared with data obtained by sample preparation with common MALDI matrices. These model molecules belonging to the class of organic molecules that have already been found in meteorite samples were selected as test compounds, as the development of the target adapter and the experiments presented in this paper represent the first step of a larger project on the direct characterization of organic compounds in meteorites in the future.

## 2 EXPERIMENTAL

### 2.1 Chemicals and materials

Analytes used for the measurements included l‐tryptophan reagent grade ≥98% HPLC (Sigma‐Aldrich, St Louis, MO, USA), 2‐deoxy‐d‐ribose ≥99.0% TLC (Sigma‐Aldrich, Steinheim, Germany) and triphenylene ≥98.0% HPLC (Fluka, Buchs, Switzerland). As MALDI matrices, 2,5‐dihydroxybenzoic acid 98% (DHB), 2,4,6‐trihydroxyacetophenone hydrate 98% (THAP) and sinapic acid ≥98% (SA) all purchased from Sigma‐Aldrich (Steinheim, Germany) were used. As solvents, *tert*‐butyl methyl ether ≥99% (Aldrich Chemical, Milwaukee, WI, USA), ultrapure water (UHQ water, 18.2 MΩ cm resistivity at 25°C) obtained from a Μillipore Simplicity UV apparatus (Millipore, Billerica, MA, USA), acetonitrile (ACN) ACS reag. Ph. Eur., methanol hypergrade for LC‐MS (MeOH) and acetone ACS reag. Ph. Eur. (all from Merck, Darmstadt, Germany) were used. Red phosphorus ≥97.0% (Sigma‐Aldrich, St Louis, MO, USA) was used for mass calibration of the intermediate‐pressure MALDI/LDI instrument. Some sample preparations were doped with sodium chloride pA from Merck.

### 2.2 Preparation of samples and solutions

The analyte solutions for the LDI and MALDI experiments were prepared in an identical fashion. Tryptophan and 2‐deoxy‐d‐ribose were both dissolved in UHQ water (2 mg/mL) and doped with 10 μL of a 100 mg/mL NaCl solution. Triphenylene was dissolved in *tert*‐butyl methyl ether (2 mg/mL). All solutions were then treated in an ultrasonic bath at maximum power for 3 min. For LDI measurements, 0.5 μL of the sample solution was deposited onto the target and left to dry at room temperature. For MALDI experiments, three matrices were used and prepared as follows: 10 mg of DHB and 1 mg of NaCl dissolved in 1 mL of H_2_O/ACN (70:30 v/v); 10 mg of THAP and 1 mg of NaCl dissolved in 1 mL of MeOH; and 5 mg of SA and 10 mg of NaCl dissolved in 1 mL of H_2_O/ACN (40:60 v/v). Sample and matrix solutions were deposited 1:1 (v/v; 0.5 μL each) on the target and dried at room temperature. For the calibration of the Axima TOF^2^ and ultrafleXtreme mass spectrometers, 0.5 μL of SA matrix solution as described above was deposited on the target plate. Calibration of the Synapt G2 mass spectrometer was performed using a solution of red phosphorus in acetone (3 mg/mL) for the LDI and MALDI measurements. Because this mixture was a suspension it was necessary to deposit 5 × 0.5 μL of the mixture on the target plate, allowing the mixture to dry between each deposition to form a homogeneous layer.

### 2.3 Stainless steel target adapter

The sample solutions, calibration solutions and MALDI matrix solutions were deposited onto a standard metallic MALDI target plate (M880675CD1; Waters, Wilmslow, UK). As this target only fits into the Waters Synapt G2 instrument, it was necessary to develop a target adapter, which would allow insertion of the Waters target into the two other instruments that were from Shimadzu and Bruker. For that purpose an MTP MSP ID 2711000091 (Bruker Daltonics, Bremen, Germany) target adapter, originally designed for holding a standard microscope slide, was modified by us so that the MALDI target plate supplied by Waters could be inserted into the target adapter obtained from Bruker. This Bruker‐based adapter also allows the use of the Waters target for the Shimadzu instrument.

**Figure 1:**
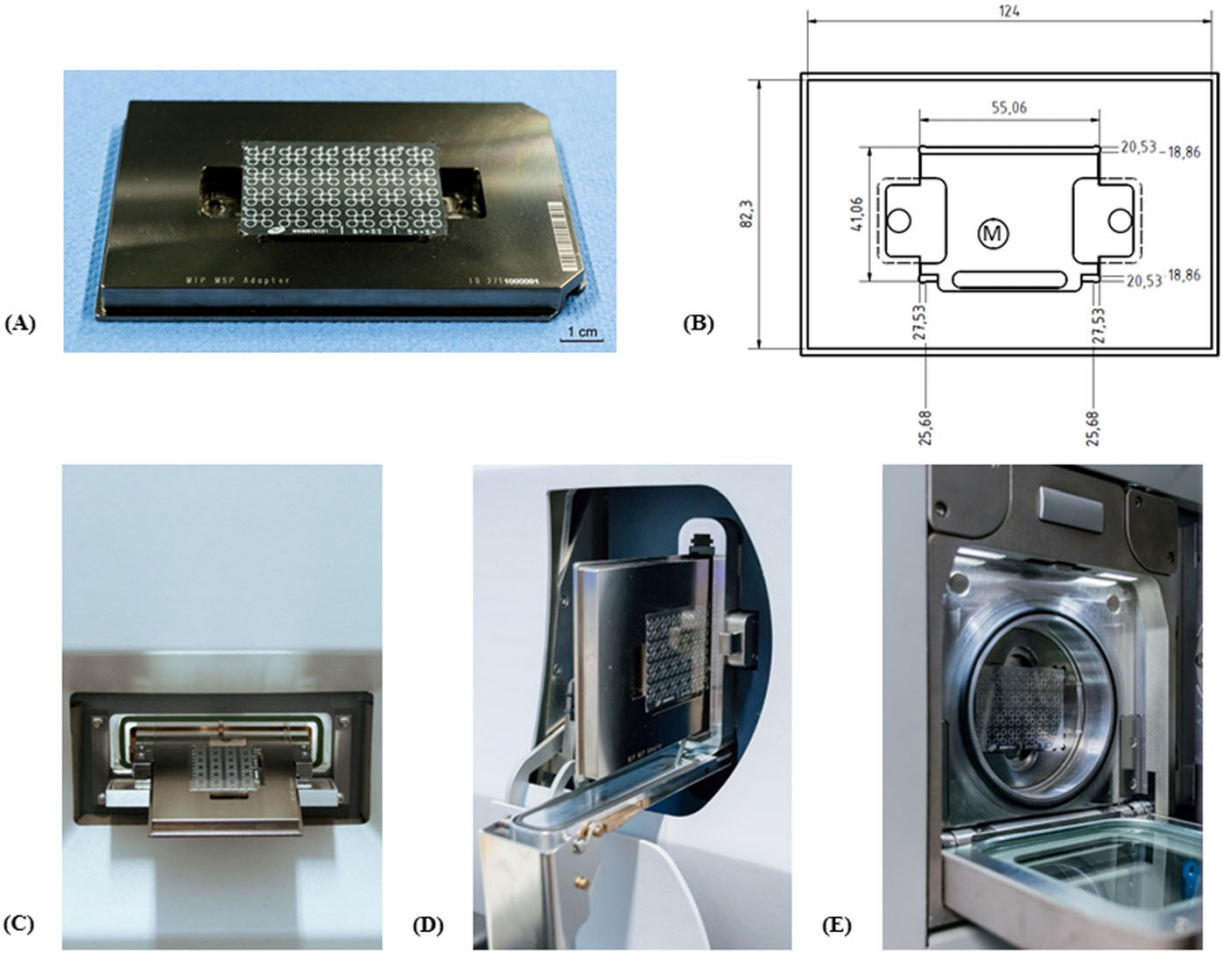
(A) Modified MALDI target (from Bruker Daltonics) with the standard MALDI target plate (from Waters) forming the target system. (B) Blueprint of the target adapter system. ‘M’ stands for magnetic disc fixing the target plate. (C) Target system with the LDI/MALDI target plate being inserted horizontally into the Axima TOF^2^ instrument. (D) Target system with the LDI/MALDI target plate being introduced vertically into the ultrafleXtreme instrument. (E) LDI/MALDI target plate being introduced into the Synapt G2 vertically without being mounted in the modified target adapter [Color figure can be viewed at http://wileyonlinelibrary.com]

The target adapter was modified by using a CNC (computer numerical control) milling machine and is shown in Figure 1A with the Waters LDI/MALDI target. The dimensions of the target adapter with the modifications are given in Figure 1B.

The LDI/MALDI target is inserted from the top into the modified target adapter/MTP target and is held there by a cylindrical magnet mounted in the target adapter cavity (Figure 1B, central circle marked with M) for insertion of the target. The target adapter can then be inserted either horizontally into the Shimadzu Axima TOF^2^ instrument or vertically into the Bruker ultrafleXtreme mass spectrometer (Figures 1C and 1D). As mentioned above, because the LDI/MALDI target plate originates from Waters, there is no need for the target adapter when using the Waters instrument, i.e. the target can be removed easily and put in the holder of the Waters Synapt G2 system. With the Waters instrument the target is inserted vertically into the ion source (Figure 1E).

The LDI/MALDI target was examined by means of the absolute digimatic indicator ID‐C125B (Mitutoyo, Kawasaki, Japan, measurement accuracy of 3 μm) to evaluate any thickness variations. No differences in the thickness of the target were observed within the measurement accuracy of the instrument. A coordinate measuring machine (Crysta‐Plus M544, Mitutoyo) was used to measure the flatness (GD&T) of the target without the target adapter as well as when the target was mounted into the adapter system. After the instrument calibration with parallel jaws (Helios‐Preisser, Gammertingen, Germany, measurement accuracy of 4 μm), the flatness of the target was 21 μm. After inserting the target into the target adapter system the overall flatness increased to 24 μm due to the adaptation of the target adapter by CNC milling. For these measurements, eight measuring points were used across the target alone and the same number of measuring points was used when the target was fixed in the adapter system by means of the small, central magnet.

### 2.4 Mass spectrometry

LDI and MALDI experiments were performed using all three instruments: the Axima TOF^2^ (Shimadzu Kratos Analytical, Manchester, UK), the ultrafleXtreme (Bruker Daltonics) and the Synapt G2 (Waters). The Axima TOF^2^ and the ultrafleXtreme are high‐vacuum instruments whereas the Synapt G2 works at intermediate pressure. For all three instruments the selected analytes were measured in positive ion mode except for tryptophan, which was measured in both positive and negative ion mode on all three instruments.

#### 2.4.1 Shimadzu Kratos Analytical Axima TOF^2^

The high‐vacuum (1.8 × 10^−7^ mBar) Axima TOF^2^ instrument is equipped with a 337 nm wavelength nitrogen laser (Gaussian beam profile) with a repetition rate of 20 Hz. This instrument is a tandem TOF mass spectrometer, which consists of a linear TOF system (MS‐1) and a wide energy acceptance (for product ions) curved field reflectron TOF as MS‐2 with an accelerating voltage of 20 kV, a differentially pumped grounded 20 keV gas collision cell (collision gas: helium) in front of the reflectron and a double Bradbury–Nielsen wire ion gate for precursor ion selection in front of the collision cell for tandem TOF experiments. LDI and MALDI experiments were performed using Launchpad version 2.8.4 MALDI‐MS software supplied by Shimadzu Kratos Analytical. All measurements were performed in positive ion reflectron and negative ion linear mode. Data were acquired in the *m*/*z* range of 20 to 500. For every measurement, 1024 single‐shot sample profiles were acquired, which equals the number of raster points over an area of 1000 μm × 1000 μm. The spacing between raster points was 32 μm. The laser fluence was adjusted for every measurement individually. Smoothing was performed by the Savitzky–Golay algorithm with a filter width of five channels. Calibration of the Axima TOF^2^ was carried out using a mixture of SA and sodium chloride by spotting 0.5 μL of matrix solution onto the target plate and performing the measurements under the same settings as described above.

#### 2.4.2 Bruker Daltonics ultrafleXtreme

The high‐vacuum (2.1 × 10^−6^ mBar) LDI/MALDI ultrafleXtreme mass spectrometer uses a 2 kHz, so‐called smartbeam–II, laser (near‐rectangular beam profile), which is a frequency‐tripled Nd:YAG laser with a wavelength of 355 nm. It is a tandem TOF instrument with a linear TOF system (MS‐1) and a dual stage reflectron as MS‐2 with a grounded 8 keV gas collision cell (collision gas: argon) immediately after the ion optics and an ion gate for precursor ion selection in front of the LIFT™ cell for tandem TOF experiments. In order to compensate for the low energy acceptance of dual‐stage reflectrons a LIFT™ cell is located between MS‐1 and MS‐2 for setting a second acceleration pulse of ±19 kV in order to ‘lift’ all product ions to a kinetic energy such that the ions are within the 30% energy acceptance of the dual‐stage reflectron. The experiments were carried out using an auto execute method from flexControl version 3.4 software and the resulting mass spectra were analysed with flexAnalysis Version 3.4 (both Bruker Daltonics). Data were acquired in the *m*/*z* range 20 to 500. The method was optimized in order to cover an area of 1000 μm × 1000 μm with 10 × 10 raster spots. Ten laser shots per raster spot were fired yielding 1000 laser shots per experiment. The applied laser fluence was adjusted manually for each experiment. Again, a mixture of SA and sodium chloride was used for the calibration of the ultrafleXtreme. A volume of 0.5 μL of matrix solution was spotted onto the target plate and measured using an automatic acquisition method developed for the samples. All measurements were performed in positive ion reflectron and negative ion reflectron modes.

#### 2.4.3 Waters Synapt G2

Intermediate‐pressure (2.1 × 10^−1^ mBar) LDI and MALDI experiments were performed with a Waters Synapt G2 QRTOF mass spectrometer equipped with a 1 kHz Nd:YAG laser operated at 355 nm (Gaussian beam profile). This instrument is a highly complex hybrid quadrupole‐reflectron TOF mass spectrometer equipped with a high‐mass transmission quadrupole, a low‐energy collision cell (*E*_Lab_ max. 100 eV), and a so‐called triwave device for ion mobility experiments. It further allows accurate mass determination with a resolution up to *R*_FWHM_ = 20 000 in the single reflectron mode (V‐mode). For acquiring and processing data, MassLynx version 4.1 software (Waters) was used. The mass range for data acquisition was set at *m*/*z* 20–500. The instrument was, as mentioned above, calibrated with red phosphorus, which was also used to perform accurate mass correction by means of the Waters lock mass system. The lock mass was acquired from the sample well for 60 s and the data were corrected with the detected lock mass at *m*/*z* 216.8163 (corresponding to the phosphorus cluster ions [P_7_]^+^ and [P_7_]^−^, neglecting the electron deficiency) automatically. Choosing the proper *m*/*z* value for the lock mass on the Synapt G2 was important to obtain accurate masses with a low deviation value. The closer the selected *m*/*z* value of the lock mass to the *m*/*z* value of the ion of interest, the higher is the achieved accuracy. The scan rate was set to 1 s per scan and the instrument mode was selected, either positive or negative ion mode. The laser fluence was set to 350 arbitrary units (au; maximum 500 au) and a spiral acquisition mode was used. The total acquisition time for one run was 120 s.

## 3 RESULTS AND DISCUSSION

All samples were measured using only one target with one preparation run for all three instruments. The target plate carrying the sample spots was inserted into the target adapter. The insertion of the target adapter carrying the LDI/MALDI target with the dried samples into the ultrafleXtreme and the Axima TOF^2^ was performed without any obstacles. As already mentioned before, it was not necessary to use the target adapter to insert the target into the Synapt G2 since we were working with the original Waters target. All experiments were carried out with three replicates of three preparations for each of the three instruments in random fashion, ensuring the reproducibility of the data obtained.

In order to exclude contamination through the possible presence of residuals on the target surface, blank target measurements were performed with all three instruments, also in random fashion. No significant ions that could interfere with the interpretation of the data were detected.

The MALDI experiments required testing of different matrices (DHB, THAP and SA) with the chosen sample molecules. This was necessary because of different interactions between the MALDI matrix molecules and the analyte molecules, i.e. to obtain optimum conditions for the desorption/ionization process.^10^, ^11^ Mass spectra of pure matrices were also acquired to ensure the proper assignment of the matrix‐related ions (DHB: M^+•^, [M + H]^+^, [M + Na]^+^, [M − H]^−^, fragment ions and cluster ions; THAP: [M + H]^+^, [M + Na]^+^, [M − H]^−^, fragment ions and cluster ions; and SA: [M + H]^+^, [M + Na]^+^, [M − H]^−^, fragment ions and cluster ions) in the final MALDI mass spectra.

Calculated monoisotopic values of positive and negative ions of the different types of molecule‐related ions ([M + H]^+^, [M − H]^−^, [M + Na]^+^, [M + 2Na − H]^+^ ions and M^+•^ radical cations) as well as measured values (average and monoisotopic depending on the applied instrument) including the deviation expressed in ppm of all compounds investigated are given in Table 1.

**Table 1:** Compounds used for this study with their obtained average/monoisotopic positive and negative ion *m*/*z* values utilizing the Axima TOF^2^, ultrafleXtreme and Synapt G2 mass spectrometers (in each case in the LDI and MALDI mode)^a^

Compound	Type of detected ion	Monoisotopic calculated *m*/*z* value	Measured *m*/*z* value					
			Axima TOF^2^		UltrafleXtreme		Synapt G2	
			LDI	MALDI	LDI	MALDI	LDI (Δ*m*, ppm)	MALDI (Δ*m*, ppm)
Tryptophan	[M + H]^+^	205.0977	205.1	205.2	ND	ND	ND	ND
	[M + Na]^+^	227.0796	227.1	227.2	227.1	227.1	227.0790 (−2.6)	227.0794 (−0.9)
	[M – H + 2Na]^+^	249.0616	249.1	249.2	249.0	249.1	249.0616 (0)	249.0613 (−1.2)
	[M − H]^−^	203.0820	203.1	203.1	203.1	203.1	203.0820 (0)	203.0815 (−2.5)
2‐Deoxy‐d‐ribose	[M + Na]^+^	157.0477	157.0	157.1	157.0	157.1	157.0479 (1.3)	157.0477 (0)
Triphenylene	[M]^+•^	228.0939	228.2	228.2	228.1	228.1	228.0930 (−3.9)	228.0936 (−1.3)

a The errors in mass determination expressed in ppm (achieved with the Synapt G2 device) are shown in parentheses. ND: not detected.

### 3.1 Tryptophan

**Figure 2:**
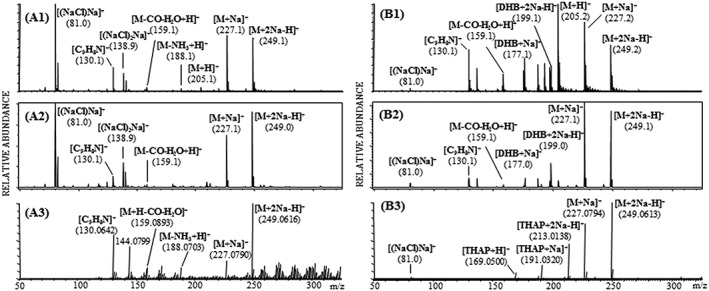
Positive‐ion LDI reflectron mass spectra of tryptophan utilizing the Axima TOF^2^ (A1), the ultrafleXtreme (A2) and the Synapt G2 (A3) instruments and positive‐ion MALDI reflectron mass spectra obtained with the Axima TOF^2^ (B1), the ultrafleXtreme (B2) and the Synapt G2 (B3) using the MALDI matrices DHB and THAP, respectively

Applying LDI and MALDI in positive ion mode, the [M + H]^+^ ion of tryptophan was detected at *m*/*z* 205.1 with the Axima TOF^2^ instrument, whereas with the ultrafleXtreme and the Synapt G2 the ion for the protonated molecule could not be detected. The MALDI matrix of choice for the analysis performed with the ultrafleXtreme and the Axima TOF^2^ was DHB, while THAP turned out to be the most suitable matrix (in terms of ion yield of analyte ions) for the analysis with the Synapt G2. The [M + Na]^+^ and [M + 2Na − H]^+^ adduct ions of tryptophan were detected at *m*/*z* 227.1 and *m*/*z* 249.1, respectively, with all three instruments by LDI as well as by MALDI (Figure 2).

**Figure 3:**
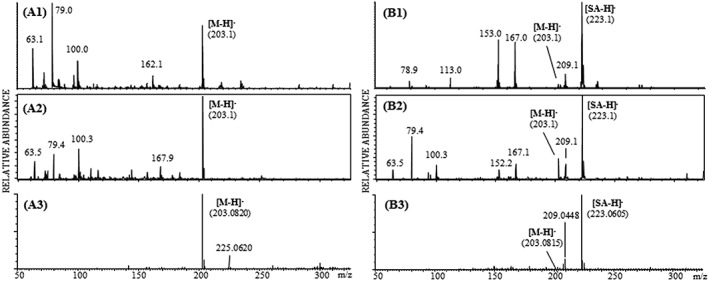
Negative‐ion LDI reflectron mass spectra of tryptophan utilizing the Axima TOF^2^ (A1), the ultrafleXtreme (A2) and the Synapt G2 (A3) instruments and negative‐ion MALDI reflectron mass spectra of tryptophan obtained with the Axima TOF^2^ (B1), the ultrafleXtreme (B2) and the Synapt G2 (B3) using the SA as MALDI matrix for all three instruments

In negative ion mode the [M − H]^−^ ion was detected at *m*/*z* 203.1 using all three instruments by LDI and with less abundance by MALDI. Due to the very abundant SA matrix signal at *m*/*z* 223.1 in negative ion MALDI mode the [M − H]^−^ ion of tryptophan seems to be either suppressed or obscured by the chemical background noise (Figure 3).

**Scheme 1:**
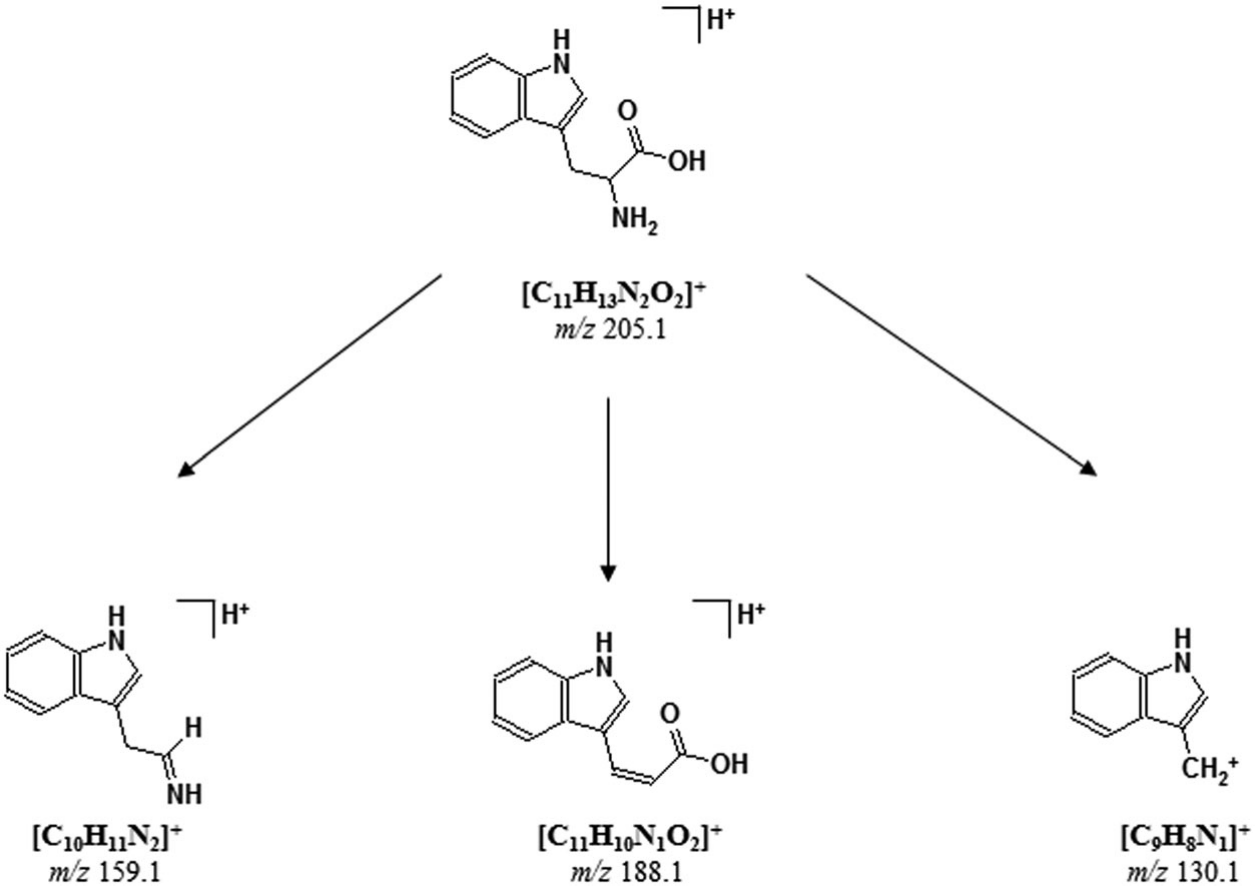
Structures of intact tryptophan ion and LDI/MALDI‐produced in‐source fragment ions at *m*/*z* 188.1, 159.1 and 130.1

In‐source‐generated fragment ions of the [M + H]^+^ ion of tryptophan are detected at *m*/*z* 130 (3‐indolylmethyl cation), *m*/*z* 159 (immonium ion of tryptophan) and *m*/*z* 188 (neutral loss of ammonia), as described by various authors.^12^, ^13^ The two fragment ions at *m*/*z* 159 and *m*/*z* 188 could be observed in positive ion mode by both LDI and MALDI. The fragment ion at *m*/*z* 130 could be observed with all three instruments during LDI whereas this particular fragment ion was found only in MALDI spectra obtained with the Axima TOF^2^ and the ultrafleXtreme. The structures of the in‐source‐produced fragment ions of the [M + H]^+^ ion of tryptophan are displayed in Scheme 1.

Other ions detected probably arise as a consequence of photochemical reactions. Sodium chloride‐spiked samples exhibit significant sodium chloride cluster ions at *m*/*z* 81 and *m*/*z* 83 corresponding to [(NaCl)Na]^+^ and *m*/*z* 139 and *m*/*z* 141 corresponding to [(NaCl)_2_Na]^+^.

The accurate mass measurements performed by the Synapt G2 include the [M + Na]^+^, [M + 2Na − H]^+^ and [M − H]^−^ ions, and the achieved accuracy is listed in Table 1.

### 3.2 2‐Deoxy‐d‐ribose

**Figure 4:**
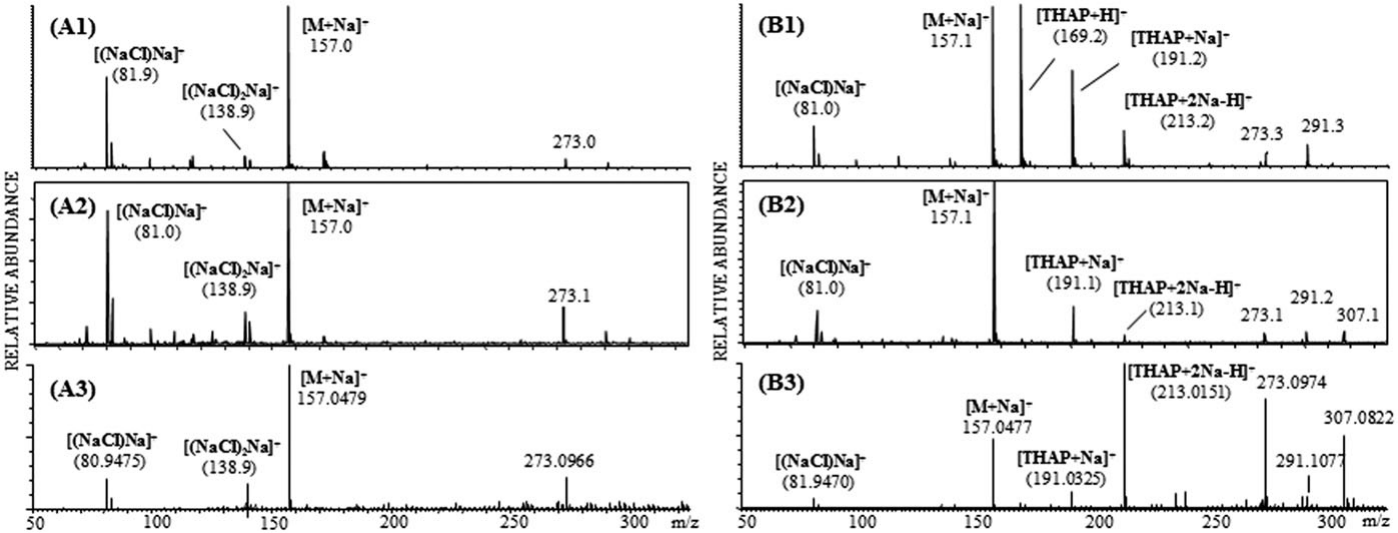
Positive‐ion LDI reflectron mass spectra of 2‐deoxy‐d‐ribose utilizing the Axima TOF^2^ (A1), the ultrafleXtreme (A2) and the Synapt G2 (A3) instruments and positive‐ion MALDI reflectron mass spectra obtained with the Axima TOF^2^ (B1), the ultrafleXtreme (B2) and the Synapt G2 (B3) using THAP as MALDI matrix for all three instruments

Being an important component of DNA, 2‐deoxy‐d‐ribose was chosen as a representative monosaccharide. 2‐Deoxy‐d‐ribose exhibits a nearly identical desorption/ionization behaviour for all three instruments by LDI, as its [M + Na]^+^ adduct (*m*/*z* 157.0) is obtained without any detectable fragmentation. Although clearly detectable by MALDI, again as its [M + Na]^+^ adduct as expected for a monosaccharide, the observed MALDI matrix background varies considerably among the three instruments when using THAP as the MALDI matrix despite a very homogeneous looking (light microscopy, data not shown) sample preparation (Figure 4).

No [M − H]^−^ ion of 2‐deoxy‐d‐ribose was formed in either LDI or MALDI mode. Accurate mass measurements of [M + Na]^+^ were performed by LDI and MALDI using the Synapt G2 (for detailed data concerning accuracy, see Table 1). The already mentioned sodium chloride cluster ions at *m*/*z* 81 and *m*/*z* 83 corresponding to [(NaCl)Na]^+^ and *m*/*z* 139 and *m*/*z* 141 corresponding to [(NaCl)_2_Na]^+^ arising from the sodium chloride added to the analyte solution were detected using LDI. With MALDI, only ions at *m*/*z* 81 and *m*/*z* 83 could be detected and no higher cluster ions.

### 3.3 Triphenylene

**Figure 5:**
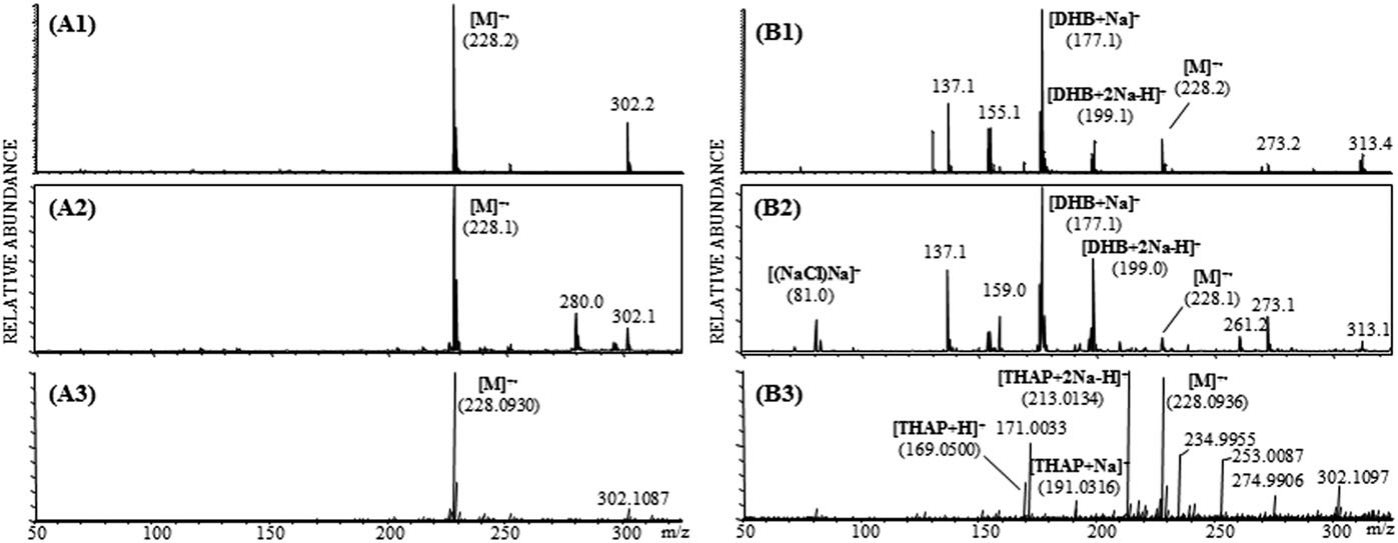
Positive‐ion LDI reflectron mass spectra of triphenylene utilizing the Axima TOF^2^ (A1), the ultrafleXtreme (A2) and the Synapt G2 (A3) instruments and positive‐ion MALDI reflectron mass spectra obtained with the Axima TOF^2^ (B1), the ultrafleXtreme (B2) and the Synapt G2 (B3) using the MALDI matrices, DHB and THAP, respectively

Triphenylene, a polycyclic aromatic hydrocarbon, was the only measured compound that did not exhibit any protonated ion, adduct ion or deprotonated ion. Only an abundant radical cation at *m*/*z* 228.1 was detected in all experimental settings. The LDI and MALDI mass spectra of triphenylene are shown in Figure 5. The observed desorption/ionization behaviour for triphenylene was nearly identical for all three instruments in LDI mode. The MALDI matrix of choice for the experiments performed with the ultrafleXtreme and Axima TOF^2^ (both high‐vacuum devices) was DHB while the best MALDI matrix for triphenylene with the Synapt G2 (intermediate‐pressure device) turned out to be THAP. This yields a significantly different matrix background (DHB versus THAP). Interestingly, the triphenylene radical cation yields the most abundant signal compared with the MALDI matrix ion when using THAP. The mass accuracies obtained for the triphenylene radical cation are displayed in Table 1.

## 4 CONCLUSIONS

A Bruker target was modified to function as an adapter for use with a standard Waters LDI/MALDI target plate. This device allowed comparison of data acquired using three different designed and performing mass spectrometers (Axima TOF^2^, ultrafleXtreme and Synapt G2) from one target plate, i.e. preparation. These instruments were typically operated under different vacuum regimes (high vacuum versus intermediate pressure), with different attached lasers (nitrogen laser versus Nd:YAG laser) at different wavelengths (337 nm versus 355 nm) and with different laser repetition rates (20 Hz versus 1 kHz versus 2 kHz). The three selected compounds (tryptophan, 2‐deoxy‐d‐ribose and triphenylene) were successfully measured with all three instruments that exhibited different performance characteristics. The mass spectrometric data obtained for tryptophan (detected as [M + Na]^+^, [M + 2Na − H]^+^ and [M − H]^−^ ions), 2‐deoxy‐d‐ribose (detected as [M + Na]^+^ ion) and triphenylene (detected as [M]^+•^ ion) were practically identical with the Axima TOF^2^, the ultrafleXtreme and the Synapt G2 in the LDI mode despite different laser wavelengths, beam shapes and repetition rates. Comparing data obtained by LDI with data obtained by MALDI, we could show that low mass MALDI matrix‐related ions usually complicate the interpretation of the data as expected for the evaluated MALDI matrices. With the high‐accuracy QRTOF instrument, the Synapt G2, we were able to obtain a mass accuracy of less than ±4 ppm for all the *m*/*z* values of interest, allowing one to determine the elemental composition of ions in the low‐mass range. The success of all LDI measurements with the use of only one target plate represents a good basis for future LDI measurements of precious carbon‐rich meteorite samples using these three instruments with their quite different performance characteristics with only a single sample preparation spot. Future investigations of carbonaceous chondrites with respect to various classes of organic molecules could help to understand the nature of meteorites^14^, ^15^, ^16^ and their influence on the development of organics on earth.
